# Team debriefing in the COVID-19 pandemic: a qualitative study of a hospital-wide clinical event debriefing program and a novel qualitative model to analyze debriefing content

**DOI:** 10.1186/s41077-022-00226-z

**Published:** 2022-10-27

**Authors:** Thomas B. Welch-Horan, Paul C. Mullan, Zobiya Momin, Jeannie Eggers, Julia B. Lawrence, Royanne L. Lichliter, Cara B. Doughty

**Affiliations:** 1 Texas Children’s Hospital, Baylor College of Medicine, 6621 Fannin St, Houston, TX 77030 USA; 2 Children’s Hospital of the King’s Daughters, Eastern Virginia Medical School, Norfolk, VA USA; 3 Children’s Healthcare of Atlanta, Emory University School of Medicine, Atlanta, GA USA; 4 Texas Children’s Hospital, Houston, TX USA

**Keywords:** Debriefing, Clinical event debriefing, Teamwork, COVID-19, Quality improvement, Patient safety, Communication, Qualitative

## Abstract

**Background:**

Healthcare workers faced unique challenges during the early months of the COVID-19 pandemic which necessitated rapid adaptation. Clinical event debriefings (CEDs) are one tool that teams can use to reflect after events and identify opportunities for improving their performance and their processes. There are few reports of how teams have used CEDs in the COVID-19 pandemic. Our aim is to explore the issues discussed during COVID-19 CEDs and propose a framework model for qualitatively analyzing CEDs.

**Methods:**

This was a descriptive, qualitative study of a hospital-wide CED program at a quaternary children’s hospital between March and July 2020. CEDs were in-person, team-led, voluntary, scripted sessions using the Debriefing in Suspected COVID-19 to Encourage Reflection and Team Learning (DISCOVER-TooL). Debriefing content was qualitatively analyzed using constant comparative coding with an integrated deductive and inductive approach. A novel conceptual framework was proposed for understanding how debriefing content can be employed at various levels in a health system for learning and improvement.

**Results:**

Thirty-one debriefings were performed and analyzed. Debriefings had a median of 7 debriefing participants, lasted a median of 10 min, and were associated with multiple systems-based process improvements. Fourteen themes and 25 subthemes were identified and categorized into a novel Input-Mediator-Output-Input Debriefing (IMOID) model. The most common themes included communication, coordination, situational awareness, team member roles, and clinical standards.

**Conclusions:**

Teams identified diverse issues in their debriefing discussions related to areas of high performance and opportunities for improvement in their care of COVID-19 patients. This model may help healthcare systems to understand how CED tools can be used to accelerate organizational learning to promote safety and improve outcomes in changing clinical environments.

**Supplementary Information:**

The online version contains supplementary material available at 10.1186/s41077-022-00226-z.

## Background

Healthcare professionals faced unique challenges while working through the early months of a global pandemic [1, 2]. The COVID-19 crisis has forced healthcare systems to rapidly respond to challenges of personal protective equipment (PPE) shortages, staff risks of exposures, patient volume surges, and evolving clinical presentations and outcomes [3–5]. The crisis has also fostered innovative approaches for teams to learn how to provide safe and effective care [6–8]. Debriefing is one of the learning tools that has been used to improve healthcare outcomes by focusing on processes that promote effective safety and teamwork practices [9, 10]. Clinical debriefings are facilitated discussions by team members about actions and thought processes with the aim of improving future performance [11, 12]. Debriefings are one example of a team reflexivity process whereby participants “reflect upon, and communicate about the group’s objectives, strategies (e.g., decision-making) and processes (e.g., communication) and adapt them to current or anticipated circumstances” [13]. In high-acuity settings, debriefings have enhanced the clinical performance of team members, informed adaptations to clinical processes, improved communication, enhanced teamwork, and supported the emotional needs of teams [12, 14–16].

Most prior reports of clinical event debriefings have focused on resuscitation events [17–20]. To help teams debrief issues related to the COVID-19 pandemic, a workgroup of healthcare providers at a quaternary children’s and women’s hospital created a debriefing tool called the Debriefing In Suspected COVid-19 to Encourage Reflection and Team Learning (DISCOVER-TooL) [21]. This novel tool was a modification of the Debriefing In-Situ Conversion after Emergent Resuscitation Now (DISCERN) tool, initially developed for “hot debriefing” (i.e., short team conversations within minutes to hours of a clinical event) in the pediatric emergency department (ED) at our hospital [18]. The aim of the DISCOVER-TooL was to help teams rapidly learn what was working well and identify opportunities for improvement that could be addressed in real time or incorporated into future process changes.

We are unaware of any studies that qualitatively describe the results of a hospital-wide, clinical event debriefing program during the COVID-19 pandemic. In this descriptive qualitative study, we present the results of COVID-19 debriefings during the initial 3 months of the pandemic.

## Methods

### Setting and study design

**Fig. 1:**
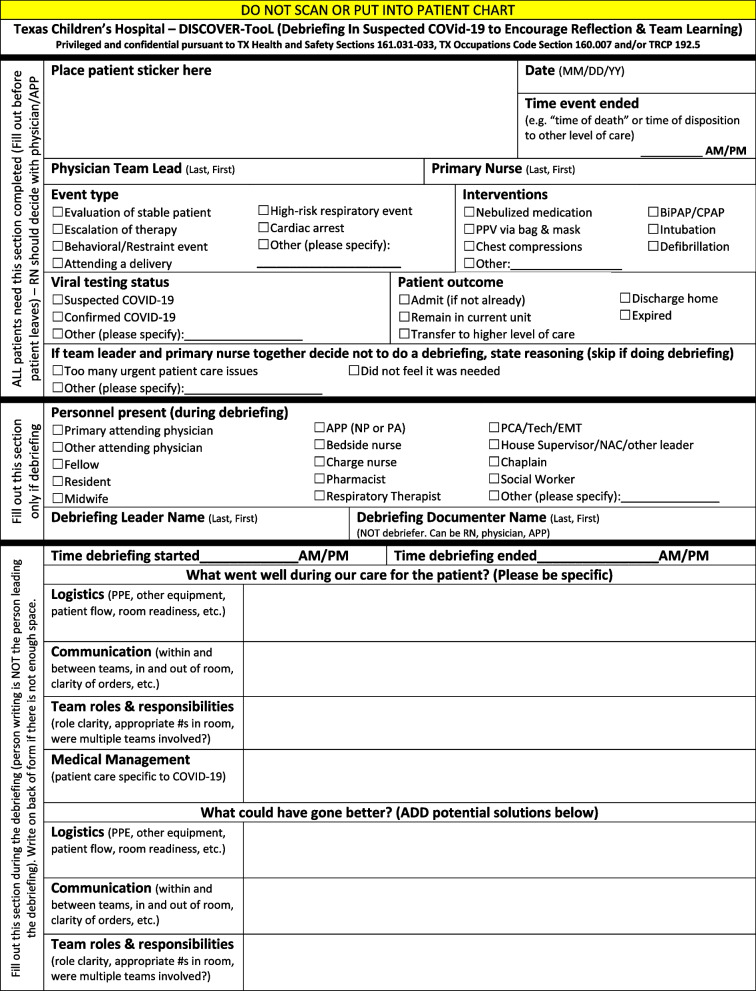
DISCOVER-TooL: Debriefing in Suspected COVID-19 to Encourage Reflection and Team Learning

**Fig. 2:**
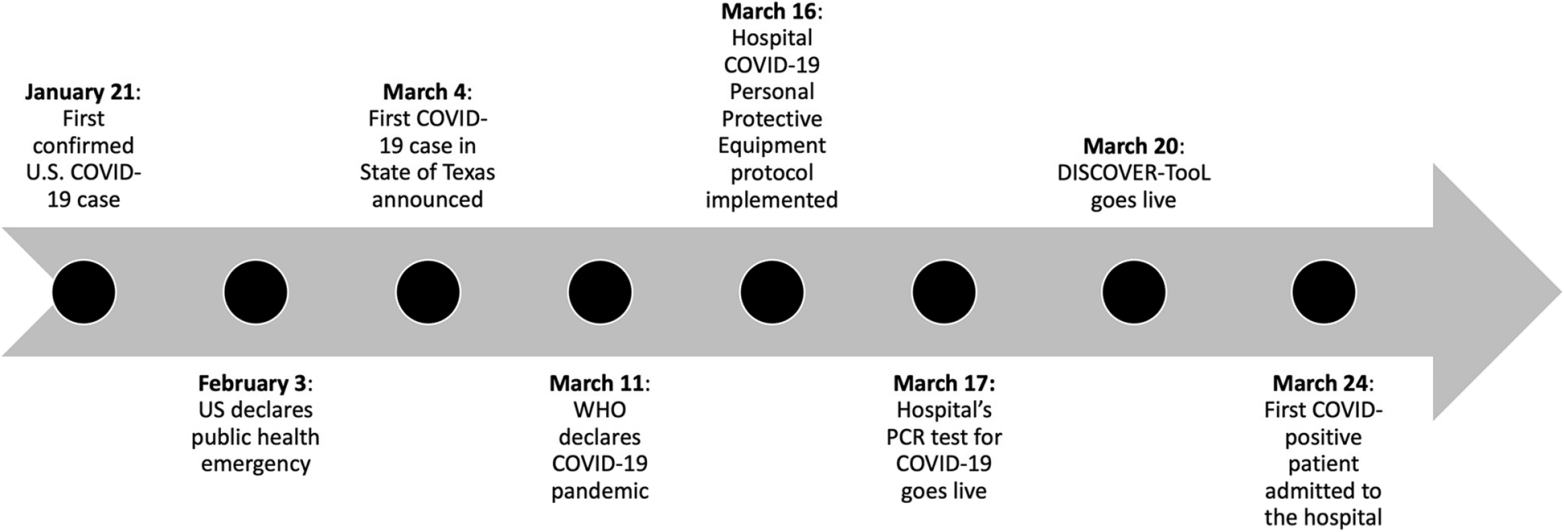
Timeline of COVID-19-related events in the hospital, state, and country during early 2020

This was a descriptive qualitative study evaluating COVID-19 clinical event debriefings using the novel DISCOVER-TooL (Fig. 1, Supplemental Figure S1) for suspected or confirmed COVID-19 patients at a quaternary care children’s and women’s hospital system from March 20, 2020 to July 31, 2020 [21]. The hospital-level timeline of COVID-19 events at our center (Fig. 2) was similar to other US-based reports [22, 23].

### Tool implementation

The DISCOVER-TooL was developed and implemented as previously described [21]. The tool was available for use in the pediatric emergency department (ED), intensive care units (ICU), acute care (i.e., inpatient non-ICU) floors, outpatient clinics, and Pavilion for Women (PFW—an obstetric and gynecologic inpatient and outpatient center). Standard descriptive statistics were used to summarize quantitative findings.

The DISCOVER-TooL workgroup informed teams throughout the hospital about the instructions for using the scripted debriefing tool via email and follow-up conversations with unit-based debriefing champions. Options for submitting the DISCOVER-Tool data included a REDCap (Vanderbilt University, Nashville, TN, USA) version (Supplemental Figure S2) or a printable version that could be emailed. The tool was also promoted in clinical units with a one-page flyer that included a website address and QR code that linked to the REDCap survey.

The workgroup reviewed survey data on a bi-weekly basis and tracked potential action items from the debriefings and referred issues to the appropriate unit-based leadership. They also identified system-wide issues and disseminated such learning opportunities to organizational leaders in meetings and/or email. The workgroup followed up with unit leaders to determine if changes to workflows, policies, or education occurred based on input from the debriefings. Selected examples of these changes were descriptively summarized. IRB approval was obtained for this study.

### Qualitative analysis

We uploaded all DISCOVER-Tool data into a Microsoft Excel database for qualitative analysis [24]. Our team had previously performed qualitative analyses and has many combined years of experience working in the acute care setting. Two investigators (TBW, PCM) initially reviewed all debriefing plus (i.e., what went well?) and delta (i.e., what could have gone better?) comments and separated out the documented debriefing comments that addressed distinct issues into quoted units for coding.

We used constant comparative coding with an integrated deductive and inductive approach [25]. We initially developed a codebook based on themes from the Team Emergency Assessment Measure framework [18, 25–27]. Two investigators (PCM, TBW) reviewed all quoted units while two investigators (CBD, ZM) coded a random subset. Themes were inductively added, iteratively adapted, or eliminated based on the coding of the debriefing comments, and the codebook was modified accordingly throughout the process. Two study investigators (PCM, TBW) discussed any discordant coding of themes until consensus was achieved. One investigator (PCM) inductively designed the conceptual model using the constant comparative method and adjusted it with workgroup feedback [25, 28].

## Results

Of the 31 debriefings in the study period, 23 had recorded locations: ED (26%), acute care (22%), PFW (22%), ICU (17%), and outpatient (13%). The debriefing duration was available for 12 debriefings (median 10.5 min [IQR 6.5, 15.5]). Of the 17 debriefings that documented the participants involved, a median of 7 (IQR 3,8) participants attended the debriefings including the nurses (100%), physicians (71%), respiratory therapists (47%), child life specialists (12%), security (12%), and pharmacists (6%). The most common debriefing facilitator was a nurse (57%) followed by a physician (43%).

**Fig. 3:**
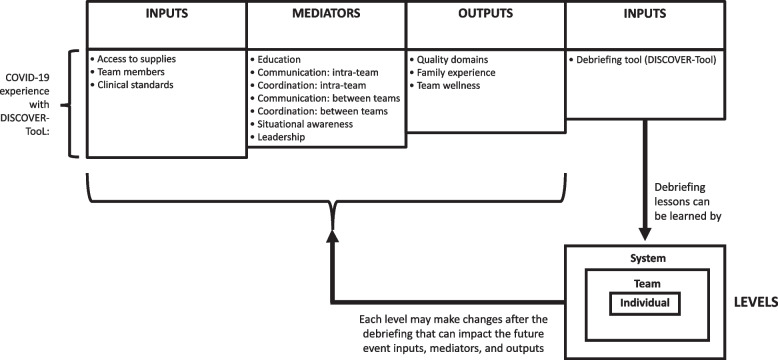
Input-mediator-output-input debriefing (IMOID) model for clinical event debriefing

All debriefings contained quoted units that were subsequently analyzed. A total of 205 quoted units from the debriefing comments were qualitatively coded (Supplemental Table S1). We identified 14 themes and 25 sub-themes and categorized these themes into a novel Input-Mediator-Output-Input Debriefing (IMOID) model with the relative frequencies of themes pictorially represented [29] (Fig. 3, Table 1). Selected examples of identified issues from debriefings that contributed to improved care were summarized (Table 2).

**Table 1:** Relative frequency of themes and subthemes in the clinical event debriefing discussions

IMOID category: Theme	Subtheme	Frequency	
		Plus comments	Delta comments
Input: Access to supplies	PPE issues	−	++
	Other equipment issues	−	++
Input: Team members	Availability of team roles	+++	++++
Input: Clinical standards	Adherence	+	+++
	Confusion or lack of awareness	−	++
	Educational opportunity	−	+
Mediator: Education	Point-of-care education	++	++
Mediator: Communication: intra-team	Acoustics and clarity	+	+
	Communication between zones	+	++
	Communication within a zone	−	++
	Documentation	−	+
Mediator: Coordination: intra-team	Minimizing team exposure opportunities	++++	++++
Mediator: Communication: between teams	Handoffs	++	+
	Multiple communications	+	++
	COVID-status communication between teams	+	+
	Under-communication	−	++
Mediator Coordination: between teams	Coordination between teams	−	+
Mediator: Situational awareness	Anticipation and readiness	++++	+
	Mental model and comprehension	+++	-
Mediator: Leadership	Leadership behaviors	+	+
Output: Quality domains	Patient centeredness, safety, timeliness, effectiveness, efficiency, and equity	*	*
Output: Family experience	Familial interactions with patient and healthcare providers	++	+
Output: Team wellness	Staff comfort	+	+
	Emotions	−	+
Input-debriefing: Debriefing experience	Debriefing process	+	−

− (Not mentioned), + (mentioned 1–4 times), ++ (mentioned 5–8 times), +++ (mentioned 9–12 times), ++++ (mentioned > 12 times)

*The quality domain frequency of comments was not specifically noted because the workgroup deemed that almost every debriefing comment made reference to one of the six Institute of Medicine Domains of Health Care Quality (safety, timeliness, patient-centeredness, efficiency, effectiveness, equity)

**Table 2:** Examples of identified issues from debriefings that were subsequently addressed to improve care processes

Situation described in debrief form	Relevant quote(s)	Clinical environment	Response from clinical team and/or leadership
The attending physician found it useful to have assistance in donning full PPE during a crisis situation	“Dress the code leader!”	Cardiac ICU	The team suggested having additional team tasks for PPE assistance and a role dedicated to helping the team leader don PPE.
The inpatient team on the COVID floor instituted a dedicated PPE doffing area and a doffing monitor team role	“EC RN unaware of the Doffing area on 15th floor ‘Covid cohort’”	General pediatric inpatient floor	Information was provided to other hospital teams regarding PPE best practices on the COVID floor as other teams were unaware of this practice.
Concerns raised by staff members about access and quality of available personal protective equipment	“RN has to make multiple calls to supply chain department to obtain N95 masks. Continued concern re: the ill fit of the blue plastic gown (mainly neckline exposure).”	Obstetrics area of the hospital (antepartum floor and/or labor and delivery floor)	The team decided to have daily huddles to review the current PPE protocol and availability. Laminated signs for reviewing PPE donning and doffing were posted in clinical areas.
During resuscitation (at a community EC site) of a patient felt to be an extracorporeal membrane oxygenation (ECMO) candidate, concerns were raised regarding which clinical team was primary, and where the patient under investigation should be cannulated	“Clarity of ‘who owns the patient, EC or PICU?’”; “Would like to have clarity about preferences of ECMO team to cannulate in EC vs PICU?”	Emergency center	Workflow regarding the preferred location for cannulation during the pandemic, and the workflow for ECMO cannulation at community sites, was reviewed with key leadership staff members.
Difficult to include members of multiple teams in the same-day debriefing of a complex event	“Debrief conducted in person and via multiple phone calls to include multiple nursing staff members from both WAC and WSU.”	Obstetrics area of the hospital	The debriefing QI team expressed appreciation of attempts to be inclusive. The debriefing workgroup recommended the usage of DISCOVER-TooL more than once per event if geographically separate teams could not simultaneously debrief.

*Abbreviations*: *EC* emergency center, i.e., emergency department, *PICU* pediatric intensive care unit, *PPE* personal protective equipment, *QI* quality improvement, *WAC* Women’s Assessment Center—an intake and triage area for pregnant women, *WSU* Women’s Specialty Unit, i.e., inpatient floor for antepartum patient’s and/or post-partum patients whose infants, because of clinical condition, are not eligible for rooming in with them

### Inputs

#### Access to supplies

Many debriefing delta comments centered on the lack of optimal access to PPE. One noted that “there were no masks in the anteroom” and another mentioned “availability of N95 masks.” Coordinating access to supplies was a challenge often, as one “charge nurse had to make multiple calls to supply chain department to obtain N95 masks.” Another debriefing noted an inability “to obtain a mask for a stat cesarean.” In other debriefings, PPE seemed to be present, but there was confusion because “[we were] not sure where the PPE was located,” or in another instance, “I had to ask multiple times where supplies were.” When PPE was accessible, one debriefing noted that “nursing staff [was] unaware that the unit was responsible for supplying respiratory therapists with N95 masks.”

#### Team members

To mitigate the infectious transmission risks to team members, debriefing discussions reinforced having “minimal staff in room.” When these processes worked well, plus comments noted “excellent crowd control” and “it was not too crowded to move around for patient care.” One team noted opportunities to decrease their exposures: “Risk of exposing 2–3 extra providers… that might have not needed to be exposed—this could’ve been discussed and arranged better. Minimize people in the room is key. Plan ahead roles better.” Similarly, another team noted: “Only one nurse to grab supplies for both nursing needs and patient—which could result in spread of contamination.” As part of flexing traditional roles, debriefing comments noted that “[it] helped to have nurses do vent changes to limit number of people in room” and that “staff stepped in where needed.”

While flexing of roles was common, debriefing comments described the need to have the right people present with their assigned roles. Role assignment quotes included “roles were designated ahead of time” and “roles were assigned in the huddle before patient showed up: inside/outside the room, role based on task.” In one event, the team positively noted that “roles were clear despite not having time to clarify prior to patient arrival,” highlighting the benefit of consistent role assignments in daily practice. Delta comments on roles commonly cited a missing team member, as in the desire to “look into a pharmacy person outside of room to assist similar to a code team” or another comment that “more support was needed for runners outside of the operating room.” When personnel were missing, teams acknowledged their impact, such as “the lack of personnel at night delayed patient care and made it more challenging” and “the teams feel they need the PPE spotter with them—it is part of their safety routine.”

#### Clinical standards

Teams discussed the need to follow both routine and pandemic-related clinical standards. One comment noted “confusion regarding the appropriate PPE for COVID positive patient—unit staff thought that the patient should wear an N95 mask” while another had a “question as to whether an outside COVID test can be acceptable.” Some of the confusion on standards varied across team roles for an event, as when the “surgery team [was] somewhat unaware of not being able to go in and out of the room.”

### Mediators

#### Education

Given the increased complexity of care, teams commented on the benefit of multi-modal point-of-care reminders of current policies. Teams acknowledged the need for more signage, including one that noted “the signs on wall/window of the room of how to properly proceed with PPE were very good.” Teams suggested solutions as well, citing that “signs to prevent extra people from going in the special isolation unit helped, [but] still people need reminders.” These reminders could include “more simulations” or the “review of the latest workflows with oncoming staff.” Front line coaches helped teams adhere to protocols as demonstrated by the comment that the “West Campus team was able to provide on-the-spot don/doffing training to ECMO team.” Another debriefing emphasized the benefit when a physician “walked them through donning/doffing procedures.”

#### Communication and coordination: intra-team

Teams acknowledged the importance of effective communication and coordination which was “a challenge due to …PPE” and the physical separation of team members to limit patient contact. At times, such separation functioned well, with “very good communication with all staff and roles within the room and outside.” Among the factors viewed as helping communication was when it was “not loud… not a lot of people in the room” and team members used “clear communication with critical language.” One team noted the “PPE makes it hard to hear anyone, especially for the documenter.” Compounding the ambient noise problem was that “PPE is so hard to hear through—[that] you end up yelling so others can hear.” Proposed solutions included “others… need to be silent” and that “to hear each other better in this situation, the team leader could have paused and asked for a timeout/silence so she could speak.” Novel communication methods were trialed, with mixed results, in this COVID-19 environment. Teams acknowledged that it was “difficult to communicate about such an ill patient via baby monitor” and that the wireless communication devices “were not great.” Suggestions for communicating better “to hear outside of the room with doors closed” included “alternative options…landline, iPads,” “having a radio inside and outside,” or “laminated signs and write on with dry-erase marker for ‘need meds’ or ‘need supplies.’”

#### Communication and coordination: between teams

Debriefing participants frequently commented on the impact of communication and handoffs between teams. At times, transitions of care went well with “good handoff from the transport team” or “experienced individuals helped handoff go well.” Debriefings commonly noted communication breakdowns, which created “confusion when ICU transitions care—ICU nurse adjusted epi drip without understanding what the patient was already on.” Another team advocated for the need to “work together to ensure improved handoffs and communication between specialists and sub-specialists.” The importance of communicating the COVID-19 status of patients at handoff was commonly cited. One team suggested “when ECMO page goes out, it would be helpful to put the COVID status on the call.” Another debriefing recommended “improved communication between obstetrics and neonatology regarding the COVID-19 status of expectant mothers.”

Coordination often required multiple communication events. One plus comment highlighted such communication functioning well when the “charge nurse and doctor talked to multiple people to try and move the patient faster.” At times, teams did not communicate enough as when one “team was unaware that ECMO team was en route so [the] room was not ready” or when the “obstetricians were not clear on the PPACT [perinatal palliative care team] plan yet and mom delivered precipitously.” At other times, teams expressed frustration that there were “wayyyy too many phone calls” needed to coordinate care because of the patient’s COVID status, and tallied their efforts when recalling that “admitting the patient …took > 7 h–3 calls to PHM [pediatric hospital medicine team], 2 calls to neuro, 2 calls to PICU, and 3 calls to house supervisor.” One solution offered by another team included having “1 person to communicate with [for admissions]—not multiple people.”

#### Situational awareness

Situational awareness was highlighted in many teams who noted the need to perceive important clinical elements, comprehend their meaning, and anticipate future circumstances. One team noted the benefit of perceiving patient acuity when they had “early identification that patient [was] ill… [with] good communication to get extra resources early.” Teams acknowledged the importance of anticipating resource needs with the “supplies ready at bedside, and plan in place in case intubation is not successful on first attempt.” Plus comments often described the value of “readiness” in various debriefings in statements like “N95s readily available when we decided to prepare for intubation,” and “meds were at the bedside, and epi ready by the time the team was dressed.” One activity that supported teams in their preparations was mental modeling, such as when the “doctor did a great job of summarizing the situation and providing a recap of care provided.” Other plus comments noted the merit of “opening up to team for ideas and mental modeling” while another cited that “mental modeling regarding potential adverse events …allowed them to plan ahead.”

#### Leadership

The role and activities of leaders were often cited. Leadership gave teams “a clear direction” as leaders “took control of the situation.” Given the necessary movement of leaders between patient care areas, one team noted the benefit when they had a good “transition of team lead role when [the] initial lead went to talk to parents.” Teams reflected in delta comments when leadership was lacking, as when one team needed “clarity on who owns the patient—ED or PICU?” and another team noted there were “too many cooks in the kitchen.”

### Outputs

#### Quality domains

Many debriefing comments noted the impacts of the aforementioned inputs and mediators on the outputs of team performance. Outputs included quality outputs for the patient, the family experience, and team wellness. The patient quality outputs included comments about patient-centeredness (e.g., “family very opposed to intubation…”), safety (e.g., “PPE monitor… made the nurse feel safe”), timeliness (e.g., “…this caused a 35-min delay for the patient”), effectiveness (e.g., “filter was not placed on ventilator”), efficiency (e.g., “not familiar with the layout and where supplies are located in room”), and equity (e.g., “patient’s local pharmacy did not have the medication in stock.”).

#### Family experience

Outcomes for the family commonly focused on the challenges of COVID-19 protocols that required either separation or cohorting of family members with their children. One debriefing reflected positively that the team was able to give “great updates to parents who were not able to be at bedside initially.” Speaking to the novel COVID-19 cohorting experience, one team noted that they had “inform[ed] mom prior to coming up that the mom would not be able to leave.” Teams commented on the relational difficulties that COVID-19 introduced when they noted a “gray area with how to interact with COVID-positive parents, especially when dealing with loss of a child—difficult for staff not to offer comfort especially as a caregiver.”

#### Team wellness

At times, team members commented on their own emotional experiences. One team noted that “everyone remained calm” as a plus comment. Issues related to staff wellness were also acknowledged in the value of having “another patient room designated as a place for staff to eat” and a delta comment on the challenge that “staff had to wear full PPE for long periods of time without a break.” Only one comment explicitly named the emotion when noting that the “RN, rightfully so, [was] very anxious about caring for patients - as she noted the nurses have to ‘do everything.’” Another comment alluded to psychological safety, stating that the “nurses said that they can’t make suggestions for care and do not feel heard.”

## Discussion

In this study, we described the implementation of a novel debriefing tool that healthcare teams used after clinical events in a hospital system during the early months of the COVID-19 pandemic. Our team rapidly adapted a previously established tool to meet the needs of healthcare teams during a pandemic [18, 21]. Teams from across the hospital used the tool to identify areas of high performance as well as opportunities for improvement. These plus and delta comments were communicated to unit and system leaders who were able to iteratively adjust team structures and care processes to remain resilient in the rapidly evolving pandemic environment.

Effective debriefing programs are designed to meet the varying needs and capabilities of each local setting. We are unaware of any published programs specifically designed to debrief teams after individual patient events in the COVID-19 pandemic setting. We are aware of several published COVID-19 debriefing program descriptions that included general quality improvement suggestions without a focus on individual clinical events. Two programs were facilitated by ED leadership groups in the USA that performed voluntary, virtual, post-shift, 30–60 min, emotionally focused debriefings with ED teams for the first 2 months of the first COVID-19 surge [30, 31]. Secondly, local simulation-trained debriefers in Belgium led voluntary, virtual, post-shift, 10-min clinical debriefing sessions with ED teams [32]. Lastly, a community-based team in Kenya led voluntary, in-person, 90-min debriefing sessions with inpatient clinicians during their clinical shifts for 2 weeks after Kenya’s first confirmed COVID-19 case [33]. Surveys of healthcare workers consistently cite time constraints as the primary barrier to debriefing [34–36]. By performing in-person, scripted, team-led debriefing sessions that were conducted shortly after the clinical event and typically lasted 10 min, our teams were able to identify Safety-I and Safety-II principles in a timely manner. These Safety-I and II perspectives focus on identifying the causes of events to minimize the number of things that can go wrong and maximize the number of things that can go right, respectively [37].

We identified 14 themes that emerged from the qualitative analysis of the debriefings and integrated these themes into an adapted version of the IMOI framework [29]. The authors of the IMOI framework describe three stages of team development as the forming stage (i.e., input-mediator phase), functioning stage (i.e., mediator-output phase), and the finishing stage (i.e., output-input phase), whereby the team “completes one episode in the developmental cycles and begins a new cycle” [29]. Despite the importance of the finishing stage, the IMOI authors cite the relative paucity of literature on this stage [29]. Our naming modification to the IMOI-Debriefing (IMOID) framework emphasizes debriefings as one of many inputs (e.g., huddles, root-cause-analyses) that teams might use in this final stage. Other debriefing researchers might use this IMOID framework to qualitatively analyze the content of their debriefings.

The themes that our teams identified in their debriefings are similar to prior qualitative analyses of team debriefings [18, 27, 38, 39]. Because of rapidly changing clinical demands during the pandemic, several variations of these themes were identified. Similar to other qualitative debriefing studies before and during the pandemic, communication and coordination issues were ubiquitous [18, 27, 31–33]. Unique pandemic-related communication barriers included the acoustic challenges with PPE, technical problems with communication devices, and separation of team members to prevent contamination. These barriers forced teams to innovate with ideas such as whiteboard messages and verbalized timeouts to hear critical communication. Realizing the difficulties in staying up-to-date on new COVID-related guidelines, teams discussed the need for additional roles (e.g., runners, PPE assistants) and multi-modal, point-of-care educational adjuncts (e.g., coaches, signage). These adjuncts have demonstrated effectiveness in other settings when teams are experiencing high cognitive demands during team performance [40, 41]. An elevated cognitive load can make attaining situational awareness more difficult, as it becomes harder to perceive, interpret, and anticipate the clinical situation [42, 43]. Team members applauded each other when this situational awareness was evident in pre-event preparations and shared mental models during the event. These shared mental models are a result of team cognition and enable the team “to coordinate seamlessly and adapt dynamically” [8, 44].

One attribute that successfully contributed to the implementation of the DISCOVER-TooL was our workgroup’s ability to adapt an existing post-event debriefing tool, which had been used locally for nearly a decade, to meet the needs of pandemic-specific circumstances. After clinical processes had undergone iterative changes and were relatively stable, DISCOVER-TooL usage declined in the hospital in approximately November 2020. The pandemic adaptation of the debriefing tool helped to accelerate our transition from a paper-based, post-event debriefing tool to an electronic version which teams now use to regularly debrief themselves on clinical matters including, but not limited to, COVID-19 issues. In future pandemics or other related crises, this tool could be further adapted to address the needs of clinical teams.

Our teams continue to participate in voluntary post-event debriefings because of the benefits relating to quality, education, teamwork, and emotional processing [10, 18]. This teamwork benefit is hard to measure, but when teams recognize the plus comments about their performance in debriefing, they likely build collective efficacy, a “belief about your team’s likelihood to successfully perform specific tasks under the current circumstances” [8]. Team debriefings can also support open, psychologically safe environments where comments can be shared that acknowledge the circumstances contributing to undesired outcomes [45]. Psychological safety in teams is one of the “strongest predictors of team effectiveness” and is fundamental to achieving organizational learning [8, 46]. Building and maintaining psychological safety will be critical for organizations that wish to reflect openly now and in the aftermath of the pandemic [8].

This study had several limitations. Although debriefings occurred across multiple units, the single academic center study design might limit the generalizability of the findings. Additionally, this medical center had a culture of debriefing, with some areas of the hospital having performed clinical event debriefings for more than a decade [18]. The adoption of similar tools in other settings, and the reflective discussions that these debriefings elicit, would likely differ across settings based on prior debriefing practices. DISCOVER-TooL was scripted in its format to provide a standard structure, build psychological safety, emphasize its intention for primarily quality and safety-related purposes, and guide participants to potentially relevant topic areas (e.g., logistics, communication, and team roles). While we intended this structure to foster team reflexivity, it is possible that the tool might have limited the discussion in other areas [13, 47]. Such a limitation could potentially explain the paucity of comments related to the emotional reactions of participants. However, the shared experiences of the authors are that such emotional reactions do occur but are not often documented. The inability of a team member to document every word of a 10-min discussion, while simultaneously facilitating or co-facilitating the discussion, is another inherent limitation to capturing the richness of the team’s discussions in qualitative analyses. Lastly, our study design could not analyze the reasons that certain teams chose not to debrief clinical events. Future qualitative focus group studies could explore such reasons and any potential downsides to the debriefing program.

## Conclusions

We believe that our analyses have important implications as teams seek to improve their collective performance using debriefing during a pandemic or in other high-stakes environments. The IMOID model outlines a framework for the role of debriefings as an input that can enable individual, team, and systems-based learning in order to reinforce future improvements. The COVID-19 pandemic disrupted some of the ways that we provide care to patients, as well as our interactions with each other and our patients. Clinical event debriefings are one way for teams to resiliently respond in these uncertain times, and as we move toward establishing “the new normal” [8]. Future steps in debriefing research should include determining the most effective methods for implementing debriefing programs and for translating data from debriefings into actionable information that can drive changes in care processes and team practices. We hope that by re-formulating data from debriefings into an input for broader change strategies, healthcare teams can use these learning conversations to make improvements for their patients and team members.

## Supplementary Information

**Additional file 1: Supplemental Figure S1.** The second page (printed on the back of the front page) of the DISCOVER-TooL.

**Additional file 2: Supplemental Figure S2.** The REDCap version of the DISCOVER-TooL questions.

**Additional file 3: Supplemental Table 1.** Extracted units from debriefing discussions that were coded into the themes.

## Data Availability

Supplemental Table 1 provides data of quoted units from the debriefings as described in the methods. The entirety of debriefing cannot be shared due concerns regarding protected health information.

## Abbreviations

CED: Clinical event debriefing
COVID-19: Coronavirus disease 2019
DISCOVER-TooL: Debriefing in Suspected COVID-19 to Encourage Reflection & Team Learning
ECMO: Extracorporeal membrane oxygenation
EC: Emergency center
ED: Emergency department
ICU: Intensive care unit
IMOID: Input-mediator-output-input debriefing
IQR: Inter-quartile range
PICU: Pediatric intensive care unit
PFW: Pavilion For Women
PPE: Personal Protective Equipment
QR: Quick response
U.S.: United States
WAC: Women’s Assessment Center
WSU: Women’s Specialty Unit
