# Supplementary material for: Team debriefing in the COVID-19 pandemic: a qualitative study of a hospital-wide clinical event debriefing program and a novel qualitative model to analyze debriefing content
Source: Adv Simul (Lond). 2022 Oct 27;7:36. doi: 10.1186/s41077-022-00226-z (PMC9612619; doi:10.1186/s41077-022-00226-z)
Supplement: Supplementary file 1 — Additional file 1: Supplemental Figure S1. The second page (printed on the back of the front page) of the DISCOVER-TooL. [file 41077_2022_226_MOESM1_ESM.pdf]

	**Medical Management**<br>(patient care specific to COVID-19)	
	**Potential solutions for improvement (Please be specific)**	

Advice for running a team debriefing

1. Pick a quiet or isolated space, if possible. Start by thanking team members for being present, and encouraging all teammates to participate.
2. State: "The purpose of debriefing is for education, quality improvement, and emotional processing. It is not a blaming session. Everyone's participation is welcome and encouraged."
3. State: "These debriefings usually take up to 10 minutes, and if you have urgent issues to attend to, you are welcome to leave at any time."
4. Begin with a reactions phase: "In one or two words, can you describe how this event made you feel?"
5. State: "Now, we will briefly review the patient's summary, and then we as an entire team can discuss what went well and what could have gone better. Please feel free to ask any questions."
6. Have team lead and/or recorder proceed with a brief summary of the patient's clinical course (<1 minute), and then proceed to the group discussion as outlined. Documenter (not person leading the debriefing) records on this form.

Co-debriefing and debriefing process improvement

1. If using a co-debriefer, have a co-debriefing plan prior to starting the debriefing session in order to stay organized and professional.
2. Reflect after the session with your co-debriefer and/or recorder on what went well, and how to improve the debriefing process next time.
3. For further guidance on use of this tool, please contact Bram Welch-Horan (tbwelchh@texaschildrens.org), Cara Doughty (cbdought@texaschildrens.org), or Cassidy Penn (cvpenn@texaschildrens.org).

Employee Resources

COVID-19 Incident Command at TCH: 832-824-0137
Support Statement: "We know these are challenging times for healthcare workers. We can take time now to discuss how people are feeling, and to remind teammates of counseling support that is available through TCH or BCM."
Anyone who needs or requests referral for free **counseling** can call the appropriate institution.

833-327-7587 (TCH EAP Plus offsite appointments); 832-824-3327 (on-site, for TCH employees only)
713-500-3008 (BCM EAP)

Instructions for filling out this form

1. If you wish to **complete the form electronically**, please use your phone to scan the QR code below and complete the REDCap survey. Click "Submit" once finished.

(QR code linking to online version of DISCOVER-TooL wasIncluded here)

2. If you prefer to **fill out the form manually**, please scan/take photo and securely email to Arjun Dangre. To send an encrypted message (at TCH) through Outlook or OWA, include "!secure!" in the subject line. !secure! is case-sensitive and can appear anywhere in the subject line.

DISCOVER-TooL v1.5, 4-26-20

Acknowledgments
Concept & design: Bram Welch-Horan, Cara Doughty, Dan Lemke. Layout, data management, logistics: Cassidy Penn, Arjun Dangre, Jeannie Eggers. Assistance and feedback: Susan Leong-Kee, Patricia Bastero, Mona Khattab, Kasey Davis, Paul Mullan, Demian Szyld, Jean-Christophe Servotte. Adapted from DISCERN tool: Mullan PC, et al. *Resuscitation* 2013;84(7):946-51.
