# Supplementary material for: Team debriefing in the COVID-19 pandemic: a qualitative study of a hospital-wide clinical event debriefing program and a novel qualitative model to analyze debriefing content
Source: Adv Simul (Lond). 2022 Oct 27;7:36. doi: 10.1186/s41077-022-00226-z (PMC9612619; doi:10.1186/s41077-022-00226-z)
Supplement: Supplementary file 2 — Additional file 2: Supplemental Figure S2. The REDCap version of the DISCOVER-TooL questions. [file 41077_2022_226_MOESM2_ESM.pdf]

DISCOVER-TooL Debriefing In Suspected COVid-19 to Encourage Reflection and Team Learning.

Texas Children's Hospital - DISCOVER-TooL Debriefing In Suspected COVid-19 to Encourage Reflection and Team Learning.. Privledged and Confidential pursant to TX Health and Safety Sections 161.031-033, TX Occupations Code Section 160.007 and/or TRCP 192.5

Updated 03/20/2020

ALL patients need this section completed - RN should decide with physicians/APP whether to debrief.

This section MUST BE COMPLETED before the patient leaves.

Downloadable Form

[Attachment: "DISCOVER TooL v1.3 - CP edits continued.docx"]

Encounter Type
- ◯ Patient Event (same day debrief of a single event)
- ◯ Patient Event (single event debrief at a later date)
- ◯ Shift Summary (same day single event debrief of multiple events)

Patient Last Name ______________________

Patient First Name ______________________

Patient MRN ______________________

Date ______________________

Physician Team Leader (Last, First Name) ______________________

Primary Nurse (Last, First Name) ______________________

Event Type
- ☐ Evaluation of stable patient
- ☐ Escalation of therapy
- ☐ Behavioral/restraint event
- ☐ Attending a delivery
- ☐ High-risk respiratory event
- ☐ Cardiac arrest
- ☐ Other

Other event type: ______________________

Interventions	☐ Nebulized medications<br>☐ BIPAP/CPAP<br>☐ PPV via bag and mask<br>☐ Intubation<br>☐ Chest compressions<br>☐ Defribrillation<br>☐ Other
Other intervention type:	______________________________
Viral testing status	○ Suspected COVID-19<br>○ Confirmed COVID-19<br>○ Other
Other viral testing status type:	______________________________
Patient outcome	○ Admit (if not already)<br>○ Remain in current unit<br>○ Transfer to higher level of care<br>○ Discharge home<br>○ Expired
Time event ended (e.g. "time of death" or time of disposition to other level of care)	______________________________
Did you conduct a debrief?	○ Yes<br>○ No
If team leader and nurse together decide not to do a debriefing, state reasoning:<br><br>(after answering, proceed to the end of the survey and click submit)	○ Too many urgent patient care issues to make time<br>○ Did not feel it was needed<br>○ Other reason
Other reason why a debrief was not completed:	______________________________________

Fill out the following sections ONLY IF debriefing occurs.

Members present during debriefing:

- ☐ Primary attending physician
- ☐ Other attending physician
- ☐ Fellow
- ☐ Resident
- ☐ Midwife
- ☐ APP (NP or PA)
- ☐ Bedside nurse
- ☐ Charge nurse
- ☐ Pharmacist
- ☐ Respiratory Therapist
- ☐ PCA/Tech/EMT
- ☐ NAC/House Supervisor/Other Leader
- ☐ Chaplain
- ☐ Social Work
- ☐ Other

Specify all "other" members present

____

Debrief Leader Name (Last Name, First Name):

____

Was there a co-debrief leader?

- ◯ Yes
- ◯ No

Co-debrief Leader Name (Last name, First Name):

____

Debriefing Documenter Name (Last name, First Name):
(Person writing NOT the person leading the debriefing)

(NOT the same as physician team lead; can be RN or physician or APP)

____

Fill out this section DURING the debriefing. Please be specific.

Advice for running a team debriefing
1. Pick a quiet or isolated space if possible. Start by thanking team members for being present and encouraging all members to participate.
2. State: "The purpose of debriefing is for education, quality improvement, and emotional processing. It is not a blaming session. Everyone's participation is welcome and encouraged."
3. State: "These debriefings usually take up to 10 minutes, and if you have urgent issues to attend to, you are welcome to leave at any time."
4. Begin with a reactions phase: "In one or two words, can you describe how this event made you feel?"
5. State: "Now, we will briefly review the patient's summary and then we as an entire team can discuss what went well and what could have gone better. Please feel free to ask any questions."
6. Have team lead and/or recorder proceed with a brief summary of the patient's course (< 1 minute) and then proceed to the group discussion as outlined on front of form. Documenter (not person leading the debriefing) records on this form.

Co-debriefing and debrief process improvement:
1. If using a co-debriefer, have a co-debriefing plan prior to starting the debriefing session in order to stay organized and professional.
2. Reflect after the session with your co-debriefer and/or recorder on what went well and how to improve the debriefing process next time.
3. For further guidance on use of this tool, please contact Bram Welch-Horan (tbwelchh@texaschildrens.org), Cara Doughty (cbdought@texaschildrens.org), or Cassidy Penn (cvpenn@texaschildrens.org)

Employee Resources
Support: We know these are challenging times for healthcare workers. We can take time right now to discuss how people are feeling and to remind teammates of counseling support that is available through TCH or BCM.

If anyone needs or requests referral for free counseling, call the appropriate institution.
832-824-3327 (TCH)
713-500-3327 (BCM)

Incident command: 832-824-0137

Time Debriefing Started: ______________________________

Fill out this section DURING the debriefing. The person writing should NOT be the person leading the debrief. Please be specific with your answers.

What went well during our care for the patient?

LOGISTICS (PPE, patient flow, room readiness, etc.) ________________________________

What went well during our care for the patient?

COMMUNICATION (within and between teams, in and out of room, clarity of orders, etc.) ________________________________

What went well during our care for the patient?

TEAM ROLES AND RESPONSIBILITIES (role clarity, appropriate numbers in room, were multiple teams involved) ________________________________

What went well during our care for the patient?

MEDICAL MANAGEMENT (patient care specific to COVID-19) ________________________________

What could have gone better?

LOGISTICS (PPE, patient flow, room readiness, etc.) ________________________________

What could have gone better?

COMMUNICATION (within and between teams, in and out of room, clarity of orders, etc.) ________________________________

What could have gone better?

TEAM ROLES AND RESPONSIBILITIES (role clarity, appropriate numbers in room, were multiple teams involved) ________________________________

What could have gone better?

MEDICAL MANAGEMENT (patient care specific to COVID-19) ________________________________

Potential solutions for improvement: ________________________________

If a safety issue was identified during debrief or patient encounter, has a safety scoop been completed?
- ◯ Yes
- ◯ No

Time Debriefing Ended: __________________________
