# Supplementary material for: Team debriefing in the COVID-19 pandemic: a qualitative study of a hospital-wide clinical event debriefing program and a novel qualitative model to analyze debriefing content
Source: Adv Simul (Lond). 2022 Oct 27;7:36. doi: 10.1186/s41077-022-00226-z (PMC9612619; doi:10.1186/s41077-022-00226-z)
Supplement: Supplementary file 3 — Additional file 3: Supplemental Table 1. Extracted units from debriefing discussions that were coded into the themes. [file 41077_2022_226_MOESM3_ESM.pdf]

Supplemental Table 1: Extracted units from debriefing discussions that were coded into the themes.

**IMOID: Theme**	**Plus Comments** *(Subthemes)*	**Delta Comments** *(Subthemes)*
INPUT: Access to supplies		*PPE issues:*<br>1. "There were no masks in the anteroom of 915."<br>2. "Nursing staff unaware that the unit was responsible for supplying respiratory therapist with N95 masks."<br>3. "Was not sure where the PPE was located. Saw boxes on charge nurse counter. Not familiar with the layout and where Supplies are located in room."<br>4. "Asked a nurse several times where things where and I was pointed and told over there, not specific location- I had to ask multiple times where supplies were."<br>5. "Discuss broader availability of N95 masks and filters in room."<br>6. "I was unable to obtain a mask for a stat cesarean. In an attempt to help, the resident had taken two from the front desk in order to give me one. The issue is that she took it into the OR with her, and I was unaware."<br>7. "CAPRs for CPR compressors."<br><br>*Other equipment issues:*<br>1. "Vent working properly for duration of pt use (stopped working when we went to transfer pt)"<br>2. "If we are responding to help the EC team, it would help to actually know where supplies are."<br>3. "There were a few meds not in anesthesia pack that were needed, fentanyl, midazolam, promethazine."<br>4. "Many items were not in the room (eg bedside carts) that would have helped especially with many emergency medications needed in an unstable patient."<br>5. "No transport ventilator readily available (this caused a 35 minute delay for the patient to move to the 5th floor OR."<br>6. "Staff/equipment in hallway was a hindrance to other patient care."<br>7. "Kit for supplies needed for transport should be already made up after hour or in the weekend process."

**IMOID: Theme**	**Plus Comments** ***(Subthemes)***	**Delta Comments** ***(Subthemes)***
INPUT:<br>Team members	1. “L&D Roles were designated ahead of time including circulator and transport team.”<br>2. “Good defined roles.”<br>3. “Roles were assigned in the huddle before patient showed up: inside/outside the room, role based on tasks (except chest tube/airway) & in case of code.”<br>3. “Roles and responsibilities were assigned and acknowledged. All three teams were engaged and responsive to their task.”<br>4. “Roles were understood, and staff stepped in where needed.”<br>5. “All staff huddled prior to case to discuss team roles and responsibilities.”<br>6. “With all staff knowing their roles and responsibilities allowed for very fluid movement.”<br>7. “Roles were clear despite not having time to clarify prior to patient arrival.”<br>8. “Everyone knew their role.”<br>9. “Everyone's role was fulfilled and no extraneous people.”<br>10. “Experienced charge nurses and leadership available to help shift staff to hot areas.”	1. “Look into a pharmacy person outside of room to assist (similar to a code team).”<br>2. “More support was needed for runners outside of the Operating Room.”<br>3. “Include Social Work on arrival notification.”<br>4. “Lack of personnel at night delayed patient care and made it more challenging. Had to call up help from the PICU (a person not trained to work in Special Isolation Unit).”<br>5. “Would have hoped for more people more quickly. Felt like a "Long Time" for other responders to enter.”<br>6. “Involve respiratory therapy leadership for planning staffing for these patients.”<br>7. “CV anesthesia PPE spotter had to leave for another's patient emergency: the team feels they need the PPE spotter with them -it is part of their safety routine.”<br>8. “The Neo RT was the first person to arrive.”<br>9. “CRNA doffed PPE after epidural bolus but did not lead transport team down to 5th floor OR elevators. He was not head of the bed on transport back after surgery either.”<br>10. "Have anesthesiologist present, in PPE if needed.”<br>11. “Respiratory therapist refused to enter patient room without N95 and elected to instruct the nurse and demonstrate product use via glass door between patient and anteroom.”<br>12. “Encouraged to assign roles in possible code event prior to patient arrival.”<br>13. “Code roles prior to start of shift.”<br>14. “When assigning roles include all key roles for potential decompensation.”<br>15. “It was understood that anesthesia would be outside the room for second attempt but was instead CRNA.”<br>16. “There were multiple teams involved. From charging I knew the RTs, the EC charge, pharmacy, and ICU team. The roles were not clear as who was doing what, maybe addressed before ICU arrived, but not when I was down there.”<br>17. “Would be better if there was a single communicator talking to the patient.”<br>18. “Confusion over how environmental services should work in this area.”

**IMOID: Theme**	**Plus Comments** ***(Subthemes)***	**Delta Comments** ***(Subthemes)***
INPUT: Clinical Standards	*Adherence:*<br>1. “Responded to security well. Good de-escalation techniques.”<br>2. “Viral filter, everyone had appropriate PPE. We had to bag patient initially.”<br>3. “Great collaboration, emergency center staff engaged with ECMO checklist, everyone remained calm.”<br>4. “Intubated quickly - Meds ordered for suspected diagnosis.”	*Adherence*:<br>1. “The workflow states that the patient care manager will help the surgeon with PPE.”<br>2. “Dietary sent tray with reusable versus disposable dishes/utensils, tray had to be remade causing delay feeding for patient.”<br>3. “Everything was wasted and the narcotics (remaining) instead of being returned were disposed - pharmacy was informed”<br>4. “1st Lumbar puncture failed but EC couldn't get pt to acute care like we *normally* do with well appearing febrile infant 15WestTower would not take report.”<br>5. “Antibiotics given during 2nd LP which was best we could do after one failed attempt.”<br>6. “Putting back pad on patient that is CPR in progress.”<br>7. “Filter was on bvm but filter, fell apart during bagging, filter not placed on ventilator (adult exploratory circuit needs viral filter ready).”<br>8. “There was a larger window between administration of Ativan and Haldol. But wanted to see if de-escalation tactics would work.”<br>9. “Eventually got the heparin from the omnicell (5-8 min delay).”<br><br>*Confusion or lack of awareness:*<br>1. “Confusion regarding the appropriate PPE for COVID positive patient. Unit staff thought that the patient should wear an N95 mask.”<br>2. “Question as to whether an outside covid test can be acceptable. Should this patient have been treated as a PUI until TCH test comes back negative?”<br>3. “Surgery team - somewhat unaware of not being able to go in and out of the room (cart outside the room).”<br>4. “Handed off some meds - who later wastes?”<br>5. “Share our neonatal guidelines with OB to ensure that they are clear.”<br>6. “Would like too have clarity about preferences of ECMO team to cannulate in EC vs PICU? PICU was under the assumption that cannulation was preferable to be done in PICU.”<br>7. “Lack of clear PPE guidelines for transport/ transfer between WSU/WAC or from unit to radiology or other non-nursing department.”<br>8. “EC RN unaware of the doffing area on 15th floor ‘covid cohort.’”<br><br>*Educational opportunity:*<br>1.“Respiratory Therapists mainly familiar with care of neo/pedi not Adults. Need respiratory therapists that are comfortable with Adults.”<br>2. “Need to compile list of unique drugs for these patients.”<br>3. “Pads on faster, more comfort with drawing up epi without pharmacy present.”

**IMOID: Theme**	**Plus Comments** ***(Subthemes)***	**Delta Comments** ***(Subthemes)***
MEDIATOR: Education	1. “Helpful to have Dr. Arrington there to set up patient room before patient arrived- walked them through donning/doffing procedures and what should be removed from room, how nurses should set up workstation, communication between nurses outside and inside the room.”<br>2. “Written instructions with how to put the vent in standby to limit number of providers in room.”<br>3. “The signs on wall/window of the room of how to properly proceed with PPE was very good.”<br>4. “Utilized West/Woodlands Community Campus Preparation for ECMO Cannulation prior to Kangaroo Crew Transport document.”<br>5. “West campus team was able to provide on the spot don/doffing training to ECMO team.”	1. “JIT reminders to review Dofficer Role/Responsibilities.”<br>2. “All staff and providers and physicians need to review Labor and delivery workflow for C-section.”<br>3. “Review of the latest workflows with oncoming staff during each start of each shift/huddle.”<br>4. “Consider trying more simulations regarding the transport of the patient.”<br>5. “Signs to prevent extra-people from going in the special isolation unit Legacy Tower 16th floor helped, still people need reminders.”<br>6. “Reminders about what can/cannot go into the room.”
MEDIATOR: Communication: intra-team	*Acoustics and clarity:*<br>1. “Not loud, not a lot of people in room.”<br>2. “Clear communication with critical language.”<br><br>*Communication between zones:*<br>1. “Good communication with pharmacy outside the room.”<br>2. “Very good communication with all staff and roles within the room and outside.”<br>3. “Appropriate signage/staff log was posted outside the room.”	*Acoustics and clarity:*<br>1. “To hear each other better in this situation, the team leader could have paused and asked for a time out/silence so she could speak.”<br>2. “Others would need to be silent.”<br><br>*Communication between zones:*<br>1. “Difficult to communicate about such an ill patient via baby monitor.”<br>2. “Maybe laminated signs and write on with dry erase marker, for "need meds" or "need supplies."<br>3. “Having radio inside and outside.”<br>4. “Difficult to communicate because speaker was difficult to hear. Voaltes [wireless wearable communication devices] were not great- discussed alternative options (Walkie talkies, landline, iPads).”<br>5. "Difficult for documenting RN to hear outside of the room with doors closed, even though we used Voalte phones.”<br><br>*Communication within a zone:*<br>1. “Hearing each other was a challenge due to number of staff and PPE required.”<br>2. “Hard to hear through the mask with intubation and getting liner.”<br>3. “CAPR easier to hear with - consider for team leader and other vital roles during codes.”<br>4. “PPE is so hard to hear through - you end up yelling so others can hear and it's hard to talk fast while yelling so others can hear.”<br>5. “Should have had clearer medication communication between medical team and nurses for medication decision.”<br><br>*Documentation*:<br>1. “No way to document code since computer is not in room- plan to use paper code sheets in future.”<br>2. “PPE makes it hard to hear anyone especially for the documenter.”<br>3. “Paperwork from blood went into the room - once in cannot come out.”<br>4. “Code documentation not done on narrator.”

IMOID:<br>Theme	Plus Comments<br>*(Subthemes)*	Delta Comments<br>*(Subthemes)*
MEDIATOR: Coordination: intra-team	*Minimizing team exposure opportunities:*<br>1. “Minimal staff in room.”<br>2. “Patient care was clustered with fetal monitoring schedule.”<br>3. “Mother (COVID+) was outside of the room and masked- transport team did not get within 6 feet of her.”<br>4. “Priority of protecting team was established early in care for patient.”<br>5. “Helped to have nurses do vent changes to limit number of people in room. Assigned nurses to stay in room/outside of room and worked well together to minimize amount of times nurses had to enter room.”<br>6. “Limited number of providers in room for code- excellent crowd control.”<br>7. “Right amount of EC staff for patient.”<br>8. “The amount of people was appropriate in the room. It was not too crowded to move around for patient care.”<br>9. “Staff listened to Dofficer to help prevent cross-contamination.”<br>10. “The OR was set up appropriately for a Covid positive patient.”<br>11. “Door shut.”<br>12. “PPE monitor, Unit orientation, Shift huddle.”<br>13. “PPE monitor was VERY helpful made the nurse feel safe. did not feel like she was compromised.”	*Minimizing team exposure opportunities:*<br>1. “We need to add role inside OR to set up room, and someone who knows supplies on outside to pass them through the door.”<br>2. “Limited nursing staff in the room. only one nurse to grab supplies for both nursing needs and patient- which could result in spread of contamination/delay of care.”<br>3. “RISK: exposing 2-3 extra providers (2 anesthesia plus extra CICU) that might have not needed to be exposed. This could've been discussed and arranged better. Minimize people in the room in key. Plan ahead roles better.”<br>4. “Surprise event - most staff not wearing advanced PPE and the bag valve mask filter, N95 masks not present for first 5 minutes of event.”<br>5. “Patient remained in non isolation room (ie EC) room, w/ mother entering & leaving room (likely Covid positive adult.”<br>6. “Security noted that seal was inadequate during physical altercation with patient.”<br>7. “Do not enter on outside of MAJOR treatment room.”<br>8. “Team felt they contaminated too many things along the way. No clear path in the corridor."<br>9. “Pharmacy not using full PPE and coming in with meds.”<br>10. "Transport team was in full PPE for transport but did not have a clean person to push.”<br>11. “Continued concern re: the ill fit of the blue plastic gown (mainly neckline exposure).”<br>12. “RN kept door ajar between anteroom and patient room, possible contaminating anteroom for a prolonged time frame after transferring patient to WSU. Unable to completely close outside patient room entry door when patient bed is in anteroom.”<br>13. “Facilities tried to remove biohazard bags that were not double bagged.”<br>14. “Wear N95, full face shield but in wrestling match nearly impossible to keep good N95 seal.”<br>15. “CLEAN AFTER YOURSELF: constant cleaning "behind" yourself is done less often than at other campus.”<br>16. “Do not enter on outside of Main Treatment Room.”<br>17. “Dress the code leader!!!”

**IMOID: Theme**	**Plus Comments** ***(Subthemes)***	**Delta Comments** ***(Subthemes)***
MEDIATOR: Communication: between teams	*Handoffs:*<br>1. “Good handoff from the transport team.”<br>2. “Good communication among the Neo team itself despite the conflicting reports.”<br>3. “Cardiology consult from gave direct signout to transport team when they picked up patient. Experienced individuals helped handoff go well.”<br>4. “Q/A between dayshift and nightshift.”<br>5. “Good handoff from the transport team.”<br><br>*Multiple communications*:<br>1. “Charge RN and MD talked to multiple people to try and move patient faster.”<br>2. “Bedside team communicated with surgery and ecmo team well and guided decision to canulate in OR.”<br>3. “The EC doctor gave us a clear understanding of what was going on ,what had been done, and that she wanted to intubate.”<br><br>*COVID-status communication between teams:*<br>1. “EC team had clear plan - Lab called on arrival to say COVID + from yesterday's EC visit.”	*Handoffs:*<br>1. “Will work together to ensure improved hand-offs and communication between specialists and subspecialists.”<br>2. “Confusion when ICU transitions cares. ICU RN adjusted epi drip without understanding what the patient was already on.”<br><br>*Multiple communications:*<br>1. “Would be better to have 1 person to communicate with- not multiple ppl on West Tower floor, house super/NAC, Mission control.”<br>2. “Admitting pt with transient ischemic attack but full return to baseline took >7 hours, 3 calls to PHM, 2 calls to neuro, 2 calls to PICU, and 3 calls to house supervisor. By this time the MRI we had ordered (with 4 hour delay) was scheduled to be performed and Pediatric Hospital Medicine requested MRI prior to accepting pt).”<br>3. “Wayyyy to many phone calls.”<br>4. “Women’s Specialty Unit [antepartum] Charge RN has to make multiple calls to supply chain department to obtain N95 masks.”<br>5. “Delay in transfer to Women’s Specialty Unit due to physician team coming to agreement re: plan of care, pulmonary and critical care medicine and maternal fetal medicine recommended discharge to home.”<br><br>*COVID-status communication between teams*:<br>1. “When ECMO page goes out it would be helpful to put the COVID status on the call - to alert people on what other supplies to bring.”<br>2. “Communication between OB and Neo regarding the mother's COVID-19 status.”<br>3. “Improved communication between obstetrics and neonatology regarding the COVID-19 status of expected mothers.”<br><br>*Under-communication:*<br>1. “Lack of communication around no anesthesia for this case and who was needed in the room.”<br>2. “Continuous communication with Picu.”<br>3. “16b team was unaware that ECMO team was en route so room was not ready.”<br>4. “Improve communication with transport teams that are not [our internal transport team] KC.”<br>5. “Maybe notify EDAT sooner.”<br>6. “MD providers and RN providers switch at different times – huddles might not align.”<br>7. “Confusion about the decision [for palliative care] - I think OB were not clear on the [palliative care] plan yet and mom delivered precipitously.”

IMOID: Theme	Plus Comments *(Subthemes)*	Delta Comments *(Subthemes)*
MEDIATOR Coordination: between teams.	1. “Once orders were received for transfer to WSU, transfer was expeditiously completed.”<br>2. “All personal protective equipment was available and the transition from the patient room to the OR and back to the patient room went very smoothly.”<br>3. “Brought back to room fast and smoothly.”	1. “Physician states it is slower moving patient that is COVID positive to the OR.”<br>2. “Security should lead all transports of COVID patient.”
MEDIATOR: Situational awareness	*Anticipation and readiness:*<br>1. “Anesthesia Team planned ahead of time before transfer of patient (close to 30 minutes).”<br>2. “PPE: Pulmonary and Critical Care Medicine was able to provide an extra mask for Women’s Specialty Unit Charge RN in case she needed to enter room emergently/urgently to assist primary RN. Room readiness: Stocked well.”<br>3. “N95s readily available when we decided to prepare for intubation.”<br>4. “Room, supplies ready at bedside, and plan in place in case intubation is not successful on first attempt.”<br>5. “Having IC at bedside, having pre-arranged plan (huddle happened before patient was admitted).”<br>6. “Someone was dressing the attending before she realized she needed to go into the room.”<br>7. “Meds were at the bedside, and Epi ready by the time the team was dressed.”<br>8. “Spacious surgical airway cart available.”<br>9. “Well established, 2nd MD ready outside the room.”<br>10. “Neo TLC [Transport Labor & Delivery Consultant Neonatologist] Team was ready and notified of patient.”<br>11. “Everyone was ready- OR, PICU, Radiology, IR, Pharmacy, EC staff, anesthesia, and surgical PA.”<br>12. “We didn't have a warning about this patient so didn't have time to get the room ready - though the team quickly organized despite this.”<br>13. “Process went smoothly, intubation seamless with GOAT, code call got needed staff, no equipment or supply needs unmet.”<br><br>*Mental model and comprehension*<br>1. “Doctor did a great job of summarizing the situation and providing a recap of care provided.”<br>2. “Clear direction from team lead - Early identification that patient is ill… Good communication to get extra resources early.”<br>3. “Clear picture of severity of illness prior to arrival. Team prepared for sick admission and maintained stability until he was put on ECMO.”<br>4. “Pre-emptive decision to go on ECMO while patient was somewhat stable led to the positive outcome and ability to go on ECMO without CPR.”<br>5. “She [lead physician] was clear of what was going on and what she was going to do.”<br>6. “Able to review past intubation, which informed management.”<br>7. “Opening up to team for ideas and Mental modeling.”<br>8. “Dr. did a great job of suggesting a code be called to quickly get the support staff needed.”<br>9. “Mental modeling regarding potential adverse events with this complex (CP, seizures, pleural effusion, pericardial window) allowed them to plan ahead.”	*Anticipation and readiness:*<br>1. “Prepare for possible intubation if needed, including viral filter.”
MEDIATOR: Leadership	1. “Transition of team lead role when initial lead went to talk to parents.”<br>2. “Security took control of the situation for de-escalation.”	1. “”Clarity of who “owns” the patient – EC or PICU?”<br>2. “Too many cooks in the kitchen. Too many different ppl at Main Campus giving us different answers.”<br>3. “Unclear primary physician PHM vs GI.”

**IMOID: Theme**	**Plus Comments** ***(Subthemes)***	**Delta Comments** ***(Subthemes)***
OUTPUT: Family experience	1. “Team tried to keep patient alive until parents arrived.”<br>2. “Great updates to parents who were not able to be at bedside initially.”<br>3. “Nurse did inform mom prior to coming up that the mom would not be able to leave. Mom has all the symptoms.”<br>4. “Great updates given to parents who were not able to be at bedside initially.”<br>5. “Communication with mother, who was masked inside the room, in order for her not to touch anything, she was consented over the phone seeing the people she was speaking to. A bit rushed as patient was grunting, Team spoke with the family afterwards too.”	1. “Patient’s local pharmacy did not have the medication in stock. This potentially caused the patient and her exposed (some are positive) family to enter more than one pharmacy for a medication that she needed and increased her anxiety.”<br>2. “Gray area with how to interact with COVID positive parents, especially when dealing with loss of child. Difficult for staff not to offer comfort especially as a caregiver.”<br>3. “Family very opposed to intubation- required entire focus of at least one staff member at all times.”<br>4. “Patient arrived and showed up at the wrong entrance – security let them in.”
OUTPUT: Team wellness	*Staff comfort*:<br>1. “Another patient room was designated as a place for staff to eat.”	*Staff comfort*:<br>1. “Staff had to wear full PPE for long periods of time without a break.”<br>2. “When in full PPE could swap out providers if able to give staff breaks from PPE (Q2hours).”<br>3. “Too many people in PPE prior to arrival.”<br><br>*Emotions:*<br>1. “Nurses said that they can't make suggestions for care and do not feel heard.”<br>2. “RN, rightfully so, very anxious about caring for patients - as she noted the nurses have to 'do everything'.”

**IMOID: Theme**	**Plus Comments** ***(Subthemes)***	**Delta Comments** ***(Subthemes)***
**INPUT:** Debriefing tool (DISCOVER-TooL)	1. “I liked that she would debrief on procedures and the plan during the course of care.”<br>2. “Debrief conducted in person included multiple nursing staff members from both Women’s Assessment Center/Women’s Specialty Unit and day and night shift.”<br>3. “Debrief was performed at two different times due to primary MD needing to see other patients. Meet with physicians first and then staff RNs after.”	
